# Point of Care Strategy for Rapid Diagnosis of Novel A/H1N1 Influenza Virus

**DOI:** 10.1371/journal.pone.0009215

**Published:** 2010-02-17

**Authors:** Antoine Nougairede, Laetitia Ninove, Christine Zandotti, Xavier de Lamballerie, Celine Gazin, Michel Drancourt, Bernard La Scola, Didier Raoult, Remi N. Charrel

**Affiliations:** 1 Fédération de Microbiologie, Assistance Publique - Hôpitaux de Marseille, Marseille, France; 2 Unité des Virus Emergents, UMR 190 “Emergence des pathologies virales”, Université de la Méditerranée & Institut de Recherche pour le Développement, Marseille, France; 3 Unité de Recherche sur les Maladies Infectieuses et Tropicales Emergentes UMR CNRS 6236 IRD 3R198, IFR 48, Faculté de Médecine, Université de la Méditerranée, Marseille, France; Washington University School of Medicine, United States of America

## Abstract

**Background:**

Within months of the emergence of the novel A/H1N1 pandemic influenza virus (nA/H1N1v), systematic screening for the surveillance of the pandemic was abandoned in France and in some other countries. At the end of June 2009, we implemented, for the public hospitals of Marseille, a Point Of Care (POC) strategy for rapid diagnosis of the novel A/H1N1 influenza virus, in order to maintain local surveillance and to evaluate locally the kinetics of the pandemic.

**Methodology/Principal Findings:**

Two POC laboratories, located in strategic places, were organized to receive and test samples 24 h/24. POC strategy consisted of receiving and processing naso-pharyngeal specimens in preparation for the rapid influenza diagnostic test (RIDT) and real-time RT-PCR assay (rtRT-PCR). This strategy had the theoretical capacity of processing up to 36 samples per 24 h. When the flow of samples was too high, the rtRT-PCR test was abandoned in the POC laboratories and transferred to the core virology laboratory. Confirmatory diagnosis was performed in the core virology laboratory twice a day using two distinct rtRT-PCR techniques that detect either influenza A virus or nA/N1N1v. Over a period of three months, 1974 samples were received in the POC laboratories, of which 111 were positive for nA/H1N1v. Specificity and sensitivity of RIDT were 100%, and 57.7% respectively. Positive results obtained using RIDT were transmitted to clinical practitioners in less than 2 hours. POC processed rtRT-PCR results were available within 7 hours, and rtRT-PCR confirmation within 24 hours.

**Conclusions/Significance:**

The POC strategy is of benefit, in all cases (with or without rtRT-PCR assay), because it provides continuous reception/processing of samples and reduction of the time to provide consolidated results to the clinical practitioners. We believe that implementation of the POC strategy for the largest number of suspect cases may improve the quality of patient care and our knowledge of the epidemiology of the pandemic.

## Introduction

In late April 2009, The World Health Organization (WHO) announced the emergence of a novel A/H1N1 influenza virus (nA/H1N1v). This virus spread rapidly, and after two months the WHO raised the alert level from phase 5 to phase 6 defining the first influenza pandemic of the 21st century [1]. At the beginning of the pandemic, some countries established measures to identify all possible cases, but rapidly and due to the constant increase of suspected cases, they decided to abandon the systematic screening.

In France, the initial strategy which commenced at the end of April 2009 relied on the early identification of suspect cases which were directed into the hospital system to be tested for nA/H1N1v, the positive cases being further hospitalized in an isolation ward to reduce secondary transmission. This approach provided information concerning the kinetics of the pandemic when the most positive cases were acquired abroad. The systematic screening of all suspect cases was abandoned on July 7^th^ and replaced by sentinel systems to estimate the number of cases from surveillance of certain populations and to target groups with higher risk of morbidity or mortality [2]. This cessation of laboratory-confirmed cases eliminated the possibility of a reliable estimation of the evolution of the pandemic in France. Figures varying from 28,000 (Groupes Régionaux d'Observation de la Grippe, France) to 130,000 (Réseau sentinelles, France) cases weekly were claimed during the same period depending on the source data [3]. The data gathered at the beginning of the pandemic in Europe clearly indicate that the positive predictive value of the clinical examination during examination by general practitioners or infectious disease specialists ranged between 8 and 25%, which is very low, thus justifying a laboratory-based diagnostic approach [2], [4], [5], [6]. This is a strong argument to maintain laboratory confirmation of nA/H1N1v suspect cases to provide robust epidemiological data. Even if the results are biased, the calculated proportion of laboratory-confirmed nA/H1N1v infection cases amongst suspect cases enables extrapolation to provide a reasonable estimation of the total number of cases. Because of the innate evolving nature of an epidemic/pandemic caused by a transmissible agent, such an extrapolation must be periodically updated for comparison with the kinetics of the pandemic from unambiguous data as derived from laboratory-confirmed diagnostic tests.

To maintain systematic surveillance in the public hospitals of Marseille, we organized our two POC laboratories to perform rapid diagnosis of nA/H1N1v. Here we describe the POC organization, the results of POC laboratories during three months, and the sensitivity and the specificity of the POC-based results during this period.

## Materials and Methods

### Clinical Samples

Following national regulations under the term of Biomedical Research (Loi Huriet-Sérusclat (loi 881138)), the signature at the hospital entrance office warrants that all samples done during hospitalization for diagnostic purpose are accessible for research (excluding human genetic research) without specific consent of the patient and then ethics approval was not asked for regarding the terms of the Loi Huriet-Sérusclat (loi 881138).

For each patient, specimens from both nostrils were obtained with the same Virocult swab (Virocult MW950; Medical Wive and Equipment Co.). Specimens collected were rapidly transported at room temperature for testing in the POC laboratory.

### Processing, Aliquoting, Internal Control Spiking and Rapid Influenza Diagnostic Test (RIDT)

In a class-2 biosafety cabinet, nasal swabs were resuspended in 1 mL of sterile PBS solution and transferred into a 2 mL cryotube. Two aliquots were prepared respectively for RIDT and nucleic acid extraction. The remaining material, to be tested in the core laboratory was (i) either stored at −80°C in the La Timone hospital POC laboratory, (ii) or stored at −20°C temporarily in the North hospital POC laboratory and shipped to La Timone core laboratory on dry ice every 3 hours. RIDT was performed using the Directigen EZ influenza A+B test (BD EZ Flu A+B, Becton, Dickinson and Company) according to the manufacturer's recommendations.

### RNA Extraction

One 200-µL aliquot of suspended nasal swab was added to a 2 mL tube containing 200 µl of AVL buffer and 10 µl of MS2 phage internal control and incubated for 10 minutes at room temperature for inactivation. Extracted nucleic acids were then purified using the EZ1 Virus Mini Kit v2.0 (elution volume: 90 µl) onto the EZ1 Biorobot (both from Qiagen).

### Quantitative Real Time PCR Assays

Each sample was tested by two rtRT-PCR systems: (i) a rtRT-PCR assay using SYBR Green technology targeting all influenza A viruses [7], and (ii) a rtRT-PCR assay specific for the nA/H1N1v. The latter was developed and provided by the French Influenza Reference National Center [2]. This test was used in both POC laboratories on a two-block SmartCycler system (reaction volume: 25 µl), and in the core laboratory on either an Mx3005p thermocycler (Stratagene) or an LC480 thermocycler (Roche) (reaction volume: 50 µl). rtRT-PCR reactions were performed using the SuperScript III Platinum One-Step qRT-PCR kit (Invitrogen) under the same conditions in POC and core laboratories. Standard quantities of 2X PCR Master Mix and SuperScript III RT/Platinum Taq Mix were added to the mixture reaction containing 0.8 µM of each primers (GRswH1-349Fw GAGCTAAGAGAGCAATTGA; GRswH1-601Rv GTAGATGGATGGTGAATG) and 0.2 µM of the probe (GRswH1-538Probe TTGCTGAGCTTTGGGTATGA [5′]Fam [3′]BHQ-1). A volume of 5 µl and 10 µl of total RNA was used for 25 µl and 50 µl final reaction volumes respectively. Positive controls consisted of (i) an *in-vitro* synthesized RNA transcript, and (ii) a patient-derived positive sample that was used in the core laboratory only. Reaction conditions were as follows: reverse transcription at 50°C for 15 min, initial denaturation at 95°C for 2 min, followed by 45 cycles of 95°C for 15 s, 60°C for 40 s.

## Results

### POC Laboratory Organization in Marseille

**Figure 1:**
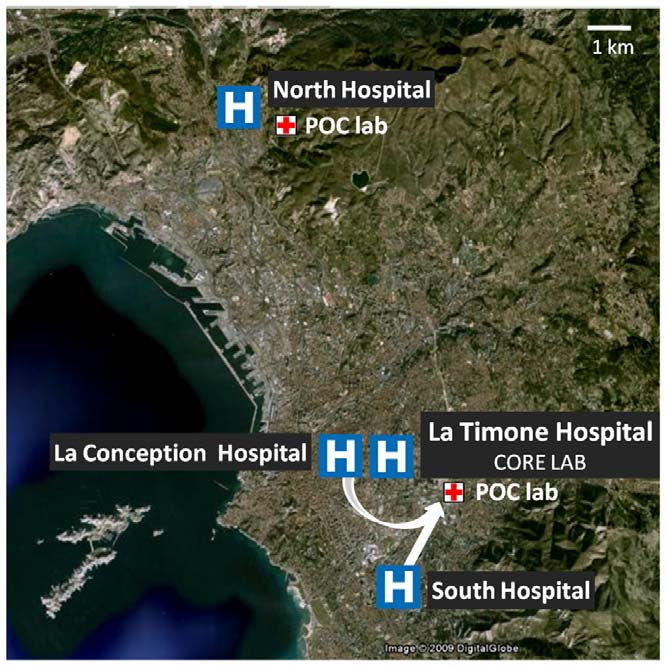
POC laboratories organization in Marseille.

In Marseille, the recent reorganization of the health structures (4 public hospitals) was achieved through the implementation of unique core laboratory for Clinical Microbiology. Centralization of the laboratories so that they serve several hospitals maximizes the efficiency of testing, thus reducing costs but has the disadvantage of poor communication with physicians in clinical wards and problems of specimen transfer [8]. As countermeasures, we decided to establish two Point Of Care (POC) laboratories located in the vicinity of the emergency units (see Figure 1) to reduce delays due to sample transportation and to reduce time to obtain the results for selected analyses. Open 24 h/24 and operated by one person, POC laboratories can rapidly perform a large panel of analyses (see Table 1). For selected parameters, the results were confirmed by the core virology laboratory.

**Table 1:** List of POC analysis.

	Pathogen agent	Specimen	Test/Target	Time for result
**Pharyngitis**	Group A Streptococcus	pharyngeal swab	ICT/Antigen	30 min
	Epstein Barr Virus	serum	ICT/Antibody	30 min
**Pneumopathy**	Mycoplasma pneumoniae	sputum or nasopharyngeal aspirate	qPCR/Genome	3 h30
	Bordetella pertussis	sputum or nasopharyngeal aspirate	qPCR/Genome	3 h30
	Legionella pneumophila	urine	ICT/Antigen	30 min
	Influenza A/B virus	nasopharyngeal aspirate	ICT/Antigen	30 min
	Respiratory syncytial virus	nasopharyngeal aspirate	ICT/Antigen	30 min
**Diarrhoea**	Rotavirus and Adenovirus	stool	ICT/Antigen	30 min
	Clostridium difficile	stool	ICT/Antigen	30 min
**Meningitis**	Neisseria meningitidis	Cerebrospinal fluid	qPCR/Genome	3 h30
	Streptococcus pneumoniae	Cerebrospinal fluid	qPCR/Genome	3 h30
	Mycoplasma pneumoniae	Cerebrospinal fluid	qPCR/Genome	3 h30
	Herpes Simplex Virus 1/2	Cerebrospinal fluid	qPCR/Genome	3 h30
	Enterovirus	Cerebrospinal fluid	qRT-PCR/genome	2 h30
	Cryptococcus	Cerebrospinal fluid	ICT/Antigen	45 min
**Gynaecology**	Group B Streptococcus	vaginal swab	qPCR/Genome	2 h30
	HIV 1/2	serum	ICT/Antibody	30 min
**Tropical fever**	Plasmodium	blood	ICT/Antigen	30 min
	Dengue Virus	serum	ICT/Antigen and Antibody	30 min
**Blood exposure accident**	HIV 1/2	serum	ICT/Antibody	30 min
**Risk of Tetanus**	Tetanus	serum	ICT/Antibody	30 min
**Gastroenterology**	Helicobacter pylori	urine	ICT/Antigen	30 min
**Urinary tract infection**	Urinary reactive strip	stool	colorimetric assay	30 min
**Procalcitonine**	Procalcitonine	serum	ICT/Antigen	45 min

ICT: immuno-chromatographic test.

### POC Laboratory Adaptation for Diagnosis of nA/H1N1v

**Figure 2:**
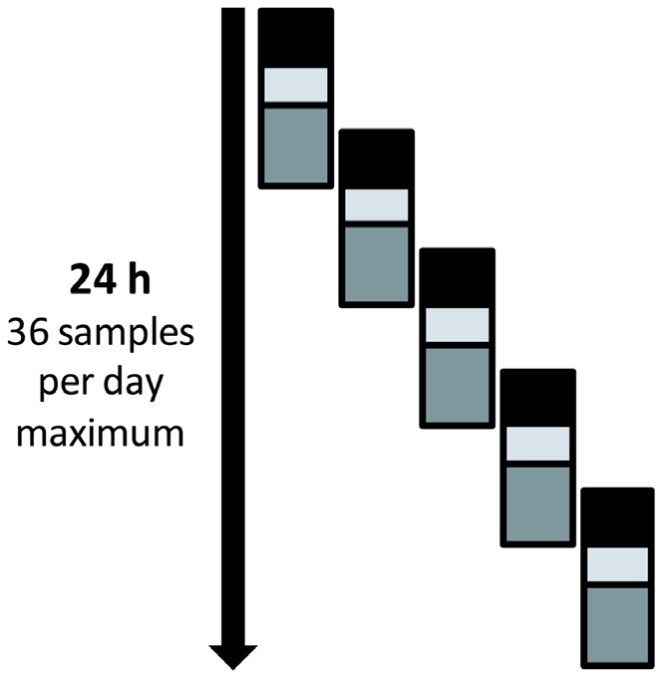
Flow capacity in POC laboratory for detection of nA/H1N1v. Black boxes represent sample receipt, registration, processing, aliquoting, internal control spiking and rapid influenza A+B test (2 h for 6 samples). Light grey boxes represent RNA extraction (6 maximum) and PCR-mix preparation (1 h30). Dark grey boxes represent rtRT-PCR (detection of nA/H1N1v on SmartCycler), interpretation and results validation (3 h).

When secondary cases of nA/H1N1v were documented in France, we decided to implement rapid detection of nA/H1N1v in the POC laboratories to provide rapid diagnosis and to study levels and variation of virus circulation. Both POC laboratories were adapted to process clinical specimens under appropriate biosafety conditions, and to perform nA/H1N1v detection via rapid influenza diagnostic tests (RIDT), and real time RT-PCR (rtRT-PCR) on SmartCycler (Cepheid). POC operators were trained to work in class II laminar hoods, and to perform RIDT and rtRT-PCR for influenza virus. The rtRT-PCR detection was performed using the test recommended by the French Influenza Reference National Centre which was adapted on SmartCycler [2]. When received at the POC laboratory, specimens were systematically spiked with an internal control that monitored all steps of the technical process from nucleic acid extraction to rtRT-PCR. Comparative evaluation with the same protocol operated in an MX3005P thermocycler (Stratagene) showed a lower sensitivity for low copy number specimens, but no significant difference in term of specificity (data not shown). Therefore positive rtRT-PCR results were validated at the POC level. The training duration for all steps of sample analysis in POC was shorter than one working day (see table 2). The cost necessary to implement POC capacity to perform nucleic acid extraction and rtRT-PCR assay was estimated at 110,000 € for the equipment and 19 € per sample (see table 2). The maximal flow capacity (upper limit) of the POC laboratory for detection of nA/H1N1v was theoretically 36 samples per day (see figure 2). In our experience, a POC laboratory was saturated when more than 20–25 samples to be tested for nA/H1N1v were received per day. This discrepancy is mainly due to two points: (i) samples were not received continuously during the 24^h^ period, (ii) POC diagnostic activities were maintained and operators had to process other samples for detection of viruses and bacteria other than nA/H1N1v (see Table 1).

**Table 2:** Equipment, cost and training duration required for POC laboratory implementation.

Equipment/assay	Cost	Maximum number of specimens tested per run	Training duration
RIDT influenza A+B (Directgen Influenza Test Kit, Becton Dickinson)	8 €/sample	-	30 min
Extraction Instrument (EZ1 BioRobot, Qiagen)	30 000 €	6 (45 min)	1 h
Viral RNA extraction (EZ1 Virus Mini Kit v2.0, Qiagen)	5 €/sample		
rtRT-PCR for nA/H1N1v and internal control (SSIII platinum one step qRT-PCR, Invitrogen)	6 €/sample	14 (2 h30)	3 h
Thermocycler (Smart cyler, Cepheid) two blocks	75 000 €		
Biosafety cabinet	≈5 000 €	-	1 h

### POC Laboratory Data from June 23^rd^ to September 27^th^ 2009

**Figure 3:**
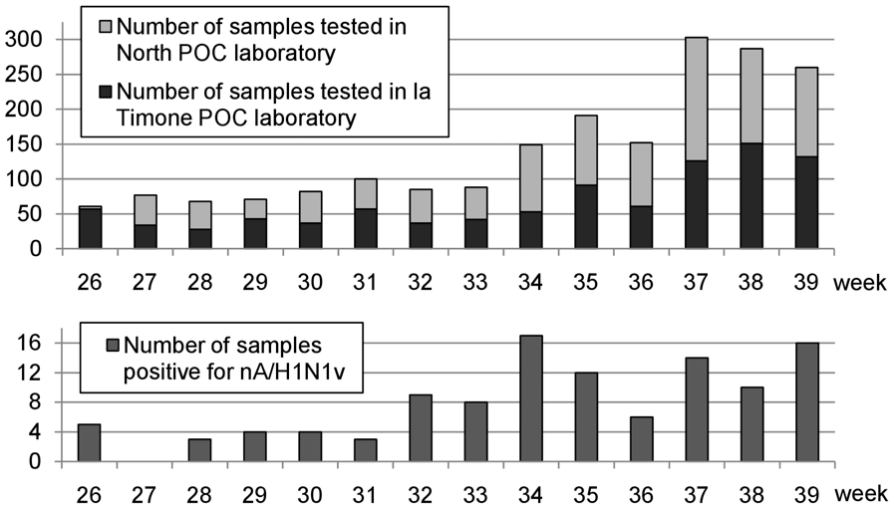
Time distribution of samples tested and positive for nA/H1N1v in POC laboratories. Positive samples correspond to rtRT-PCR core laboratory-confirmed results regardless the result obtained at the POC level.

From June 23^rd^ 2009 to September 27^th^ 2009, 1974 samples were analyzed in the two POC laboratories (see figure 3). The majority of samples originated from emergency wards (pediatrics and adults) (64.7%) and from the specific influenza consultation located in the North Hospital (9.5%). The median and mean ages were 21 years and 25.4 years respectively for these 1974 patients (see table 3). The two POC laboratories processed a similar number of specimens. At the beginning of the period, each laboratory tested about 30–40 samples per week. Then there was an obvious increase in the activity with more than 150 samples weekly from mid August to Sept 7^th^, and more than 250 samples weekly for the three last weeks of September (see figure 3). In late August, this rapid increase of clinical specimens constrained us to stop performing rtRT-PCR, while sample processing, RIDT and nucleic acid extraction were maintained at the POC level.

**Table 3:** Characteristics and results for each group described in this study.

	Age mean (year)	Age median (year)	Samples tested	Samples positive for nA/H1N1v	Samples positive for seasonal influenza virus
**All POC samples**	25.4	21	1974	111 (5.6%)	9
**Adults emergency**	42.0	36	596	28 (4.7%)	2
**Pediatrics emergency**	4.0	3	682	45 (6.6%)	1

From late June to late August 990 samples were processed in POC laboratories. During this period, a proportion of samples, for which nucleic acid extraction was performed at POC level, was rtRT-PCR tested only in the core laboratory (427 samples); 563/990 samples were tested using the rtRT-PCR on SmartCycler in POC laboratories, of which 33 (5.9%) were positive. From late August to late September, all samples extracted by POC were tested directly by the core laboratory twice a day.

**Figure 4:**
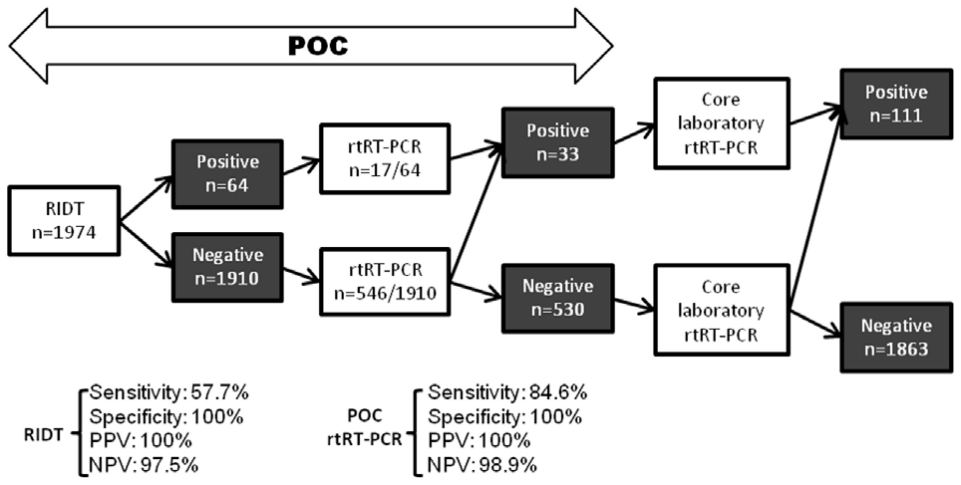
Flow chart of POC samples with sensitivity and specificity of each step. PPV: Positive predictive value. NPV: Negative predictive value.

Among the 1974 samples received in POC laboratories, 111 (5.6%) samples were positive after core laboratory confirmation using rtRT-PCR. A total of 1974 RIDT were performed, of which 64 were positive. These 64 RIDT positive samples were confirmed by A/H1N1 rtRT-PCR, then RIDT specificity was 100% (no false positive). Of the 111 core laboratory positive samples, 47 were not detected using RIDT (false negative). Therefore, the sensitivity of the POC strategy for A/H1N1 was 57.7% using RIDT and 84.6% using POC rtRT-PCR assay. We determined the positive and negative predictive value (PPV and NPV) for each step of the POC process (see figure 4).

From mid-August, the number of samples received for testing started to increase. In parallel, the number of positive samples equal to or higher than 10 per week was observed (except for week 36; see figure 3). During September, we observed an increase of specimens received for testing, whereas the number of positive samples remained stable being constantly lower than 6% of tested samples (see figure 3).

The delay necessary to obtain the results was (i) <2 h for the 64 samples with positive RIDT and (ii) 4–7 h for the 546 samples - with negative RIDT - tested using rtRT-PCR in POC laboratories. For the remaining 1364 samples, the uninterrupted nucleic acid extraction at POC level reduced the time to obtain the results (10–24 h). Computer-based communication of results also saved the time that would normally be dedicated to telephone calls for both the physician and the POC operator.

**Figure 5:**
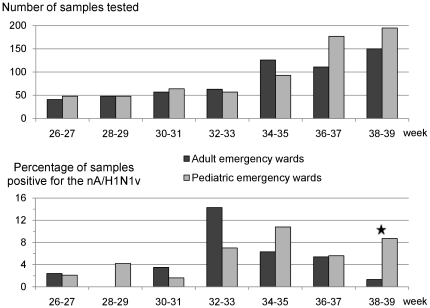
Time distribution of samples received/positive from adult and pediatric emergency wards. Positive samples correspond to rtRT-PCR core laboratory-confirmed results regardless the result obtained at the POC level. ★: p<0.01 (chi-square test).

To assess circulation of nA/H1N1v in infants and in adults, we compared the percentage of positive samples in pediatric and adult emergency wards (Table 3). From late June to mid-August, pediatric and adult emergency wards tested about 3 patients per day. The number of positive samples was low until August, then it sharply increased. In August and September, the percentage of positive samples progressively increased for children and decreased for adults, indicating a tendency that persisted in October (data not shown; figure 5).

### Core Laboratory Data from April 25^th^ to September 27^th^ 2009

**Figure 6:**
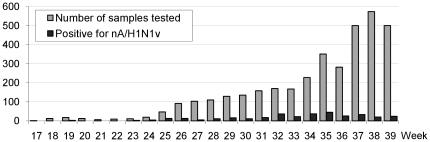
Number of samples tested/positive in the core laboratory from April to September 2009. Samples were tested using two rtRT-PCR assays (see materials and methods).

The core laboratory processed confirmatory tests for all samples received at POC laboratories using (i) the rtRT-PCR nA/H1N1v assay aforementioned and (ii) an rtRT-PCR SYBR Green system which detected all Influenza A viruses [7]. Both PCR reactions were performed on either the Mx3005p thermocycler (Stratagene) or the LC480 thermocycler (Roche). From April 25^th^ 2009 to September 27^th^ 2009, the core laboratory received and processed 3609 samples for nA/H1N1v detection. A total of 339 samples were positive (9.4%). During the entire period, our laboratory was in charge of testing human samples corresponding to 9 French departments (south-eastern France) representing 8 million inhabitants. From late April to early July, the authorities recommended to test all suspect cases [2]. During this period up to 50 specimens were received weekly. On June 23^rd^ 2009, we started to implement the POC strategy for all samples received from Marseille public hospitals, while samples received from hospitals and practitioners outside of Marseille were processed directly in the core laboratory. This strategic change was synchronous with a drastic increase in the weekly number of samples. During this period, about 100–150 samples were received each week until mid-August which witnessed an obvious increase in the activity with more than 200 samples during the next three weeks and more than 500 samples in the following three weeks of September (see figure 6).

## Discussion

POC strategy for nA/H1N1v requires minimal training, necessitates a small dedicated laboratory area available in any hospital setting and can be operated by one person. It enables the hospital to provide diagnosis within the period of clinical illness. This is imperative if one wishes to take the decision to treat the patient, to decide to isolate the patient to prevent nosocomial transmission, or to discharge the patient. It provides reliable data for studies of the local epidemiology and its evolution. It also contributes to defining and monitoring the epidemiology on a larger scale (regional, national, international), via extrapolation based on the percentage of laboratory-confirmed cases compared with suspect cases. The usefulness of the POC strategy in the context of the nA/H1N1v pandemic demonstrates that POC could usefully be implemented throughout the country not only for emergency situations but also as a daily tool to improve the quality of care for hospitalized patients, by shortening the delays and allowing decisions during the period of clinical presentation. It is likely that hospitalization costs would also directly benefit from POC strategy, through reduction of the duration of hospitalization as previously demonstrated [9], [10]. Our experience demonstrates that POC laboratory implementation reduces the time necessary to obtain the results in all cases, even when the molecular techniques are not performed at the POC level. Although, the sensitivity of POC tests is usually lower than that of rtRT-PCR techniques, their high PPV enables clinical decisions to be taken much more rapidly than in the standard diagnostic approach.

One can argue that an efficient transportation system may be less expensive than setting up a POC facility because of the short distance (less than 10 km) between the North Hospital and the core laboratory. However, our experience is that delays due to transfer for medical ward to core laboratory can vary greatly (up to 7 hours). In our hospital system, samples transportation is operated by messengers (with cars). The long delays may be sometimes due to heavy traffic between the two hospitals (more than 1 hour). Beside, as MDs, we have no hierarchical authority on the messengers. This situation has been discussed with the administrative head of the hospital, but no satisfactory solution could be found. This is the reason why the POC laboratory solution has been considered and developed.

In our opinion, the decision to abandon systematic laboratory testing of suspect patients was equivalent to breaking the thermometer while attempting to define the body temperature curve. We believe that the application of diagnostic tools for respiratory tract infections (RTI) enables the doctors and nurses to work efficiently to combat this group of diseases that the most common cause of death worldwide [11] and the most neglected cause of reduced longevity [12]. In the current pandemic situation where less than 10% of tested specimens were found positive for nA/H1N1v, the economic impact of rapid testing through the POC strategy is very important. Indeed, all negative patients - the large majority of suspect patients for the considered study period - can therefore return to their professional activities immediately. The re-admission of students in the educational course is often conditioned by a certificate assessing the absence of contagiousness for nA/H1N1v, which needs to be based on specific virologic diagnostic tests such as those implemented in the POC strategy. Finally, a better knowledge of the local and seasonal ecology of RTI microorganisms must help to determine the panel of agents to be tested at the POC level. As an example, the findings that 34% of suspected nA/H1N1v infections were in fact due to rhinoviruses [13] should theoretically justify POC testing for these viruses. It is also likely that the panel of POC detected pathogens will progressively increase to other agents causing respiratory tract infections. To address influenza morbidity and mortality, the approach based on systematic screening is more efficient than passive methods that produce underestimated data, in part due to the fact that they cannot consider the role of unrecognized influenza infection as a decisive co-morbidity factor in patients with underlying cardiovascular disease, hypertension, chronic pulmonary diseases and endocrine disorders [14]. Therefore passive surveillance tends to hinder the knowledge of epidemiology of this pandemic [3]. Systematic detection for each patient with severe influenza-associated pathology like acute respiratory distress syndrome [15] or for each patient with high mortality risk like pregnant woman [16] may contribute to appreciating the true incidence of influenza infection in such specific groups.
