# Correction: Point of Care Strategy for Rapid Diagnosis of Novel A/H1N1 Influenza Virus

**DOI:** 10.1371/annotation/5c0b32ed-3c18-4634-9f59-f5968e91710f

**Published:** 2010-04-27

**Authors:** Antoine Nougairede, Laetitia Ninove, Christine Zandotti, Xavier de Lamballerie, Celine Gazin, Michel Drancourt, Bernard La Scola, Didier Raoult, Remi N. Charrel

The following information was omitted from the funding statement:

This study has been partly funded by (i) the PHRC 2008-A00793-52, (ii) the PhleboMED project #ANR-08-MIE-023 (National Research Agency).

